# Subendocardial contractile impairment in chronic ischemic myocardium: assessment by strain analysis of 3T tagged CMR

**DOI:** 10.1186/1532-429X-14-14

**Published:** 2012-02-02

**Authors:** Michinobu Nagao, Masamitsu Hatakenaka, Yoshio Matsuo, Takeshi Kamitani, Ko Higuchi, Fumiaki Shikata, Mitsugi Nagashima, Teruhito Mochizuki, Hiroshi Honda

**Affiliations:** 1 Department of Molecular Imaging and Diagnosis, Graduate School of Medical Sciences, Kyushu University, 3-1-1 Maidashi, Higashi-Ku Fukuoka-city, Fukuoka, 812-8582, Japan; 2 Department of Clinical Radiology, Graduate School of Medical Sciences, Kyushu University, 3-1-1 Maidashi, Higashi-Ku Fukuoka-city, Fukuoka, 812-8582, Japan; 3 Department of Cardiovascular Surgery, Ehime University Graduate School of Medicine, Shitsukawa, Toon-city, Ehime, 791-0295, Japan; 4 Department of Radiology, Ehime University Graduate School of Medicine, Shitsukawa, Toon-city, Ehime, 791-0295, Japan

**Keywords:** Tagged MRI, myocardial strain, coronary artery disease

## Abstract

**Background:**

The purpose of this study was to quantify myocardial strain on the subendocardial and epicardial layers of the left ventricle (LV) using tagged cardiovascular magnetic resonance (CMR) and to investigate the transmural degree of contractile impairment in the chronic ischemic myocardium.

**Methods:**

3T tagged CMR was performed at rest in 12 patients with severe coronary artery disease who had been scheduled for coronary artery bypass grafting. Circumferential strain (C-strain) at end-systole on subendocardial and epicardial layers was measured using the short-axis tagged images of the LV and available software (Intag; Osirix). The myocardial segment was divided into stenotic and non-stenotic segments by invasive coronary angiography, and ischemic and non-ischemic segments by stress myocardial perfusion scintigraphy. The difference in C-strain between the two groups was analyzed using the Mann-Whitney U-test. The diagnostic capability of C-strain was analyzed using receiver operating characteristics analysis.

**Results:**

The absolute subendocardial C-strain was significantly lower for stenotic (-7.5 ± 12.6%) than non-stenotic segment (-18.8 ± 10.2%, p < 0.0001). There was no difference in epicardial C-strain between the two groups. Use of cutoff thresholds for subendocardial C-strain differentiated stenotic segments from non-stenotic segments with a sensitivity of 77%, a specificity of 70%, and areas under the curve (AUC) of 0.76. The absolute subendocardial C-strain was significantly lower for ischemic (-6.7 ± 13.1%) than non-ischemic segments (-21.6 ± 7.0%, p < 0.0001). The absolute epicardial C-strain was also significantly lower for ischemic (-5.1 ± 7.8%) than non-ischemic segments (-9.6 ± 9.1%, p < 0.05). Use of cutoff thresholds for subendocardial C-strain differentiated ischemic segments from non-ischemic segments with sensitivities of 86%, specificities of 84%, and AUC of 0.86.

**Conclusions:**

Analysis of tagged CMR can non-invasively demonstrate predominant impairment of subendocardial strain in the chronic ischemic myocardium at rest.

## Background

Systolic wall thickening of the left ventricle (LV) is distributed transmurally inhomogeneously. In the normal myocardium, subendocardial deformation becomes markedly greater than subepicardial deformation [1]. The progression of myocardial ischemia has a waveform appearance, which initiates at the subendocardium and extends in a gradient to the epicardial layer. The subendocardium is often the earliest myocardial layer affected in the processes of ischemia [2].

Intra-myocardial mechanisms, including wall motion strains and/or the torsion angles, can be measured when a pre-saturation tag pattern of cardiovascular magnetic resonance (CMR) is added to cine imaging. The basic principle is to tag the myocardium physically using spatially selective saturation pulses and to track the displacement of the tagged myocardium. CMR-tagging has been well demonstrated in animal studies [3-5]. Myocardial function after infarction has been studied by CMR-tagging [6-8] and a decrease in the transmural gradient of circumferential strain (C-strain) has been demonstrated in rats [7].

The purpose of this study was to quantify myocardial strain on the subendocardial and epicardial layers of LV using tagged MR imaging in patients with severe coronary artery disease (CAD) and to investigate the transmural degree of left ventricular deformation in the chronic ischemic myocardium at rest.

## Methods

### Patient population

The study protocol was reviewed and approved by the institutional review board, and written informed consent was obtained from all patients.

Seventeen patients with coronary artery stenosis, who were diagnosed as having ≥ 1 severe coronary stenosis (> 75% stenosis) using quantitative coronary artery angiography (CAG) and had been scheduled for coronary artery bypass grafting, were registered. All the patients underwent 3T tagged and late gadolinium enhancement (LGE) CMR, CAG, and stress 201-thallium myocardial perfusion SPECT within 2 weeks before revascularization therapy. The 5 of 17 patients who had LGE areas were excluded in this study. Finally, 12 patients were prospectively enrolled (Table 1). In addition, the exclusion criteria for this study were as follows: (i) acute myocardial infarction; (ii) unstable angina; (iii) a history of previous revascularization therapy (iv) greater than first degree atrioventricular block; (v) severe lower cardiac function (left ventricular ejection fraction < 20%); and (vi) New York Heart Association class IV congestive heart failure.

**Table 1:** Patient characteristics

No. of patients	12
Age (y) (mean ± SD)	70 ± 7
Gender (male/female)	8/4
BMI (mean ± SD)	23 ± 3
Risk factors	
Hypertension	10/12 (83%)
Diabetes mellitus	7/12 (58%)
Hyperlipidemia	10/12 (83%)
Smoking	6/12 (50%)
CAD	
Single-vessel disease	2/12 (17%)
Double-vessel disease	3/12 (25%)
Triple-vessel disease	7/12 (58%)
LVEF(%) (evaluated by cone MRI)	53 ± 15
(mean ± SD)	

In addition, 12 healthy volunteers (8 men, 4 women; mean age ± standard deviation, 56 ± 9 years) were enrolled as a control group. In all controls, tagged MR imaging was performed using the same protocol as for the patient group.

### CMR

All patients underwent CMR with a 3-T clinical unit (Achieva 3.0T Quarsar Dual; Philips Healthcare, Best, The Netherlands), 6- or 32-element cardiac phased-array coils were used for radiofrequency reception, and a 4-lead vector cardiogram was used for cardiac gating. After localization of the heart, 8 to 10 contiguous short-axis sections were used to cover the entire left ventricle from the base to the apex. Cine images were obtained using a balanced turbo field-echo sequence. Imaging parameters were as follows: repetition time = 3.3 msec, echo time = 1.4 msec, flip angle = 45°, section thickness = 10 mm, field of view = 380 mm, matrix size = 256 × 160, SENSE factor = 2, 20 cardiac phases/R-R interval on ECG.

**Figure 1:**
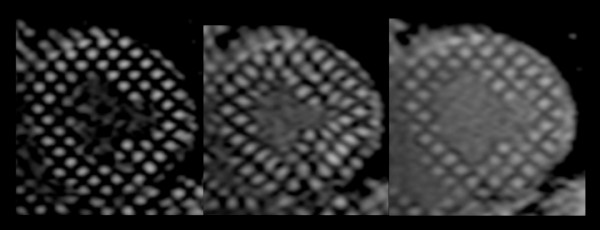
**71-year-old man with triple-vessel disease**. Short-axis mid-ventricular views of tagged cine CMR images (left: first frame at end-diastole; center: end-systole; right: last frame at end-diastole). The tagging grid persists throughout the cardiac phase on cine imaging.

Tagged CMR images were obtained in the short axis image plane of the left ventricle by using a 2D turbo field-echo sequence with a rest grid pulse. Imaging parameters were as follows: repetition time = 4.6 msec, echo time = 2.7 msec, flip angle = 12°, section thickness = 10 mm, field of view = 380 mm, matrix size = 256 × 179, SENSE factor = 2, tag grid = 6.6 mm, 20 cardiac phases/R-R interval on ECG. The mid-ventricular level of tagged CMR was used for analysis of myocardial strain (Figure 1).

### Coronary angiography (CAG)

CAG was performed by standard transfemoral arterial catheterization. A minimum of 8 projections were obtained (minimum of 5 views for the left coronary artery system and minimum of 3 views for the right coronary artery system). All CAG images were saved to CD-ROM and interpreted by two cardiologists over 10-years' clinical experience blinded to any other results (F. S. and K. K.). Coronary artery segments were evaluated using a 15-segment AHA coronary tree model. Quantitative angiographic analysis was performed on the most severe, well-defined lesion in each segment, using a previously described digital caliper method [9]. In the case of multiple lesions in a given segment, the worst lesion categorized the segment. In the cases of multiple abnormal segments per artery, the worst segment categorized the vessels. In this study, the three vessel-territories (right coronary artery: RCA, left anterior descending artery: LAD, left circumflex artery: LCX) were analyzed according to the above-mentioned form. The stenosis of the left main trunk (> 50%) was classified as the double-vessel disease (LAD and LCX).

### Stress/redistribution thallium-201 MPS

Adenosine-stress/redistribution thallium-201 MPS was performed according to the American College of Cardiology/American Heart Association/American Society of Nuclear Cardiology guidelines for the clinical use of cardiac radionuclide imaging. Pharmacological stress was induced with adenosine loading (0.16 mg/kg/min, 5 min) as described by Miyagawa et al. [10]. The adenosine-stress tests were carefully performed without concomitant anti-anginal medication and/or caffeine intake for at least 24 hours before the examination. Standard ECG, vital signs, and the general condition were continuously monitored during the stress protocol. Three minutes after the continuous infusion of adenosine, 111 MBq thallium-201 (FUJIFILM RI Pharma, Tokyo, Japan) was injected intravenously and flushed with saline. Stress images and redistribution images were acquired 10 min after the adenosine stress test and 4 h after the stress images, respectively, using a three-headed SPECT system (GCA 9300; Toshiba, Tokyo, Japan). Tomographic reconstruction was performed using a standard filtered back-projection technique with a ramp filter to produce a transaxial tomogram. No scatter or attenuation correction was applied. From these transaxial tomograms, the long axis of the left ventricle was identified and oblique-angled tomograms were generated (i.e., vertical long-axis, short-axis and horizontal long-axis tomograms). The SPECT images were visually and independently analyzed by two experienced radiologists (M. N. and T. M.). The slices were displayed sequentially to assess the myocardial perfusion in each vascular territory. The presence or absence of redistribution was visually judged in the 4 h images, which were used to determine whether ischemia was present.

### Data Analysis

**Figure 2:**
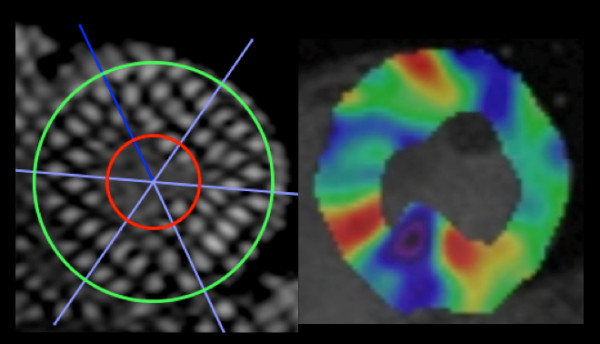
**Using inTag Osirix software for tag analysis, first we draw concentric circles of inner and outer contours of the left ventricle at end-systolic and divided the short-axis mid-ventricle into 6 segments (left)**. The software automatically plotted the area of the left ventricle and produced a color-coded strain map that shows low values as cold colors and high value as hot colors (right).

Tag analysis was performed using the open source software Osirix inTag http://www.osirix-viewer.com with the sine-wave modeling method [11]. The sine-wave model method is based on simultaneous detection of local spatial phase shift and spatial frequency in bandpass-filtered images. Image texture may be a regular grid or an irregular speckle pattern. Although tags deteriorate with time, while anatomical features penetrate the images, the sine-wave model method appears relatively robust for these changes. Within a unique environment, it integrates motion estimation in sequences of tagged images with the sine-wave modeling method, automatic extraction of the heart boundaries, and generation of deformation parameter maps that are directly exploitable by the clinician. The extraction of the heart boundaries is based on mask images computed from filtered tagged images generated in the sine-wave modeling process. Segmentation tracking is achieved by deforming the extracted contours on a reference image at mid-systole on the basis of the estimated motion fields by Sine-wave modeling. High gradients in motion help to find the external contours of the heart, as illustrated in Figure 2.

**Figure 3:**
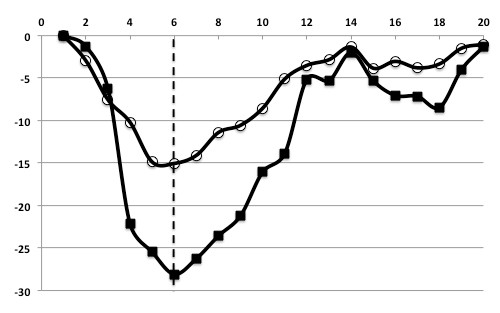
**55-year-old man healthy control**. Time curves of circumferential strain (C-strain) on an anteroseptal segment as control. The *x-axis *indicates time frames (20 frame/cycle), and the *y-axis *indicates the C-strain value (%). *Dotted line *indicates the end-systolic frame. In normal contraction, peak C-strain was a negative value. The time curve of subendocardial C-strain shows a great peak at end-systole and epicardial C-strain is relatively small. ■: subendocardial C-strain, ○: epicardial C-strain.

**Figure 4:**
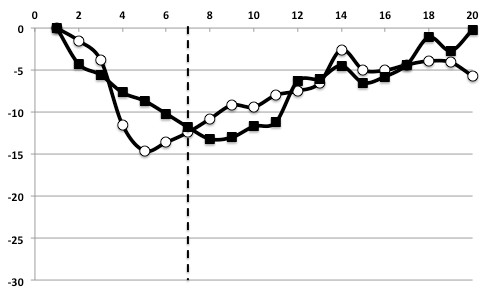
**71-year-old man with triple-vessel disease**. Time curves of circumferential strain (C-strain) on an ischemic anteroseptal segment. The *x-axis *indicates time frames (20 frame/cycle), and the *y-axis *indicates the C-strain value (%). *Dotted line *indicates the end-systolic frame. In the ischemic myocardium, the time curves of C-strain shows slow slopes and small peaks at end-systole. Subendocardial C-strain becomes similar to epicardial C-strain. ■: subendocardial C-strain, ○: epicardial C-strain.

Short-axis tagged images of the mid-LV were divided into 6 segments as follows: anterior, anteroseptal, inferoseptal, inferior, inferolateral, and anterolateral. Circumferential strain (C-strain) on the subendocardial and epicardial layer in each segment was measured during the entire cardiac cycle. At end-systole, subendocardial and epicardial C-strain was used as estimates of regional contraction (Figure 3, 4). Inferior and inferoseptal segments were identified as territories of the right coronary artery (RCA), anterior and anteroseptal segments were territories of the left anterior descending artery (LAD), and inferolateral and anterolateral were territories of the left circumflex artery (LCX). In patients with CAD, the 6 segments were categorized into ischemic and non-ischemic segments, according to the results of stress/redistribution 201-thallium SPECT.

### Statistical analysis

Continuous data are expressed as the means ± SD. The difference in the C-strain value between patients with CAD and control groups and between subendocardial and epicardial layers was analyzed using an unpaired t test. The difference in the C-strain value between the two groups of ischemic and non-ischemic, and the control segments was analyzed using the Mann-Whitney U-test. The diagnostic capability of C-strain was analyzed using receiver operating characteristics (ROC) analysis. A probability value of less than 0.05 was considered statistically significant.

## Results

**Figure 5:**
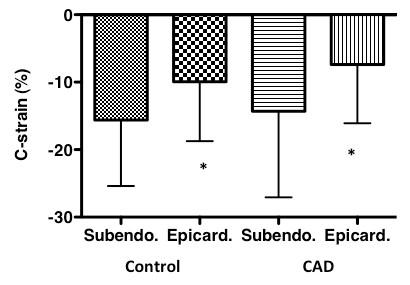
**Comparison of subendocardial and epicardial circumferential strain (C-strain) in controls and patients with coronary artery disease (CAD)**. *Bars *and *horizontal lines *indicate means and standard deviations. In controls and patients with CAD, subendocardial C-strain was significantly greater than epicardial C-strain (*p < 0.0005).

In all patients with CAD, the absolute value of C-strain was significantly greater for the subendocardial layer (-14.3 ± 12.7%) than epicardial layer (-7.4 ± 9.5%, p < 0.0005). In all controls, the absolute value of C-strain was significantly greater for the subendocardial layer (-15.6 ± 9.8%) than epicardial layer (-10.0 ± 8.8%, p < 0.0005) (Figure 5). There was no difference in subendocardial and epicardial C-strain for all segments between patients with CAD and control groups.

### C-strain vs. CAG

**Figure 6:**
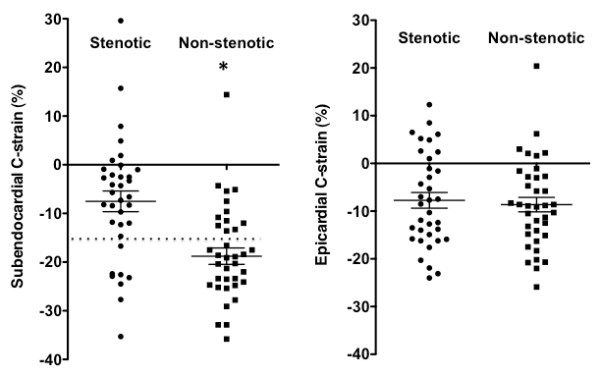
**Scatter plot shows subendocardial and epicardial circumferential strain (C-strain) in stenotic and non-stenotic segments in patients with coronary artery disease**. *Horizontal long line *represents the mean value and the *upper *and *lower short lines *the standard error of the mean. *Dotted line *represents the best cutoff threshold of C-strain. Subendocardial C-strain was significantly lower for stenotic segment than non-stenotic segment (*p <0.0001). There was no difference in epicardial C-strain between the two segments.

CAG detected 19 significant stenotic coronary arteries in 12 patients, including 4 RCA, 7 LAD, and 8 LCX. The absolute value of subendocardial C-strain was significantly lower for the stenotic segment (-7.5 ± 12.6%) than non-stenotic segment (-18.8 ± 10.2%, p < 0.0001) and the control segment (-15.6 ± 9.8%, p = 0.0006). There was no difference in subendocardial C-strain between non-stenotic and control segments. The cutoff threshold of -16.7% of subendocardial C-strain differentiated stenotic segments from non-stenotic segments with a sensitivity of 77%, a specificity of 70%, an accuracy of 74% and areas under the curve (AUC) of 0.76 (Figure 6). There was no difference in epicardial C-strain between any two groups of stenotic (-7.7 ± 9.8%), non-stenotic (-8.6 ± 9.3%) and control segments (-9.9 ± 8.8%).

### C-strain vs. SPECT

**Figure 7:**
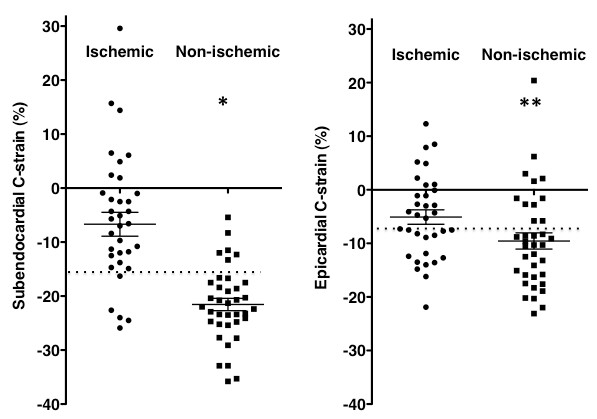
**Scatter plot shows subendocardial and epicardial circumferential strain (C-strain) in ischemic and non-ischemic segments in patients with coronary artery disease**. *Horizontal long line *represents the mean value and the *upper *and *lower short lines *the standard error of the mean. *Dotted line *represents the best cutoff threshold of C-strain. Both subendocardial and epicardial C-strains were significantly lower in ischemic than non-ischemic segments (*p < 0.0001, **p < 0.05).

Stress/redistribution MPS detected 35 ischemic segments (49%) out of 72 segments in 11 of 12 patients, including 14 segments in the LAD region, 13 segments in the LCX region and 8 segments in the RCA region. The absolute value of subendocardial C-strain was significantly lower for the ischemic segment (-6.7 ± 13.1%) than non-ischemic segment (-21.6 ± 7.0%, p < 0.0001) and the control segment (p < 0.0005), and was significantly greater for the non-ischemic than control segment (p < 0.05). The cutoff threshold of -16.3% of subendocardial C-strain differentiated ischemic segments from non-ischemic segments with a sensitivity of 86%, a specificity of 84%, an accuracy of 85% and AUC of 0.86 (Figure 7). The absolute value of epicardial C-strain was significantly lower for the ischemic (-5.1 ± 7.8%) than non-ischemic segment (-9.6 ± 9.1%, p < 0.05) and control segment (p < 0.01). There was no difference in epicardial C-strain between non-ischemic and control segments. The cutoff threshold of -8.5% of epicardial C-strain differentiated ischemic segments from non-ischemic segments with a sensitivity of 71%, a specificity of 67%, an accuracy of 69% and AUC of 0.68 (Figure 7).

### CAG vs. SPECT

Using the results of CAG as standard reference, the diagnostic capability of SPECT was sensitivity of 77%, specificity of 78%, and accuracy of 78%.

## Discussion

Strain analysis of 3T tagged MR has enabled the quantification of transmural heterogeneity in myocardial systolic mechanics in vivo in the human body. Ours is the non-invasive analytical technique for assessing subendocardial and epicardial layer strains on LV using tagged MR. In patients with severe CAD in the resting condition, subendocardial C-strain in the stenotic coronary segments was significantly lower than non-stenotic segments, whereas there was no difference in epicardial C-strain between the two segments. Diagnostic capability of subendocardial C-strain was sensitivity of 77%, specificity of 70%, and accuracy of 74%, and was about equal to that of SPECT. Furthermore, subendocardial C-strain significantly decreased in the ischemic myocardium depicted by SPECT. The subendocardium is vulnerable to change early in the process of ischemia due to several factors; it is the furthest layer from epicardial coronary flow, it undergoes extreme fluctuations in pressure and compression in both systole and diastole, and also appears prone to early structural microvascular architectural change [2]. Our results demonstrated predominant impairment of subendocardial strain induced by ischemia present at rest. Subendocardial C-strain is a more sensitive index for the detection of ischemia than epicardial C-strain.

Subendocardial C-strain for non-ischemic segments in patients with CAD was significantly greater than controls. In addition, subendocardial C-strain for non-stenotic segment tended to be greater than controls. These suggest that the decreased circumferential shortening induced in ischemic segments is compensated for by the augmentation of circumferential strain in non-ischemic segments. This compensatory mechanism allows the preservation of overall LVEF, as indicating the mean LVEF of 53%.

In the comparison of subendocardial and epicardial C-strain, subendocardial C-strain was significantly greater than epicardial C-strain in both patients with CAD and controls. Normal contraction is heterogeneous, with subendocardial deformation being markedly greater than subepicardial deformation [1]. Our results agree with the transmural gradient of myocardial strain from previous animal studies [12,13] and recent works using the speckle-tracking method of echocardiography [14,15]. In normal contraction of the heart, the free wall of the right ventricle contracts predominately in the meridian direction, whereas the free wall of LV contracts predominately in the circumferential direction so the interventricular septum makes a deformation between them [16]. This mechanical characteristic may be related to variations in circumferential shortening, resulting in a large variation by each segment in C-strain value.

The accuracy of strain calculation depends mainly on the ability to isolate the first harmonic spectral peaks, and correctly estimate HARP images [17]. The T1 relaxation time of 3-T is longer than that for 1.5-T, so the tagging grid persisted throughout the entire cardiac cycle using a 3-T scanner. Visualization of the tagging grid in the low-noise environment of 3-T was superior to that of 1.5-T [18,19]. 3-T scanners might contribute to reducing the effect of the phase wrapping function and to correctly isolate the first harmonic spectral peaks. Recently, tagged CMR analysis has used for the mechanical characteristics of location at different distances from the infarct and revealed that the reduction of myocardial strain extended well beyond the infarction to the adjacent area [20].

This study had several limitations. Subendocardial deformation is greatest in the longitudinal plane, with both electrical and mechanical activation at this level propagating from apex to base [21]; however, longitudinal myocardial strain was not calculated in our analysis. In addition, a 6.6-mm tag grid was used in this study. The half-thickness of the myocardial wall occasionally becomes less than 5 mm during the cardiac cycle. When half of the LV wall is thinner than the tagging grid size, systolic deformation in the radial direction for inner and outer layers might contain error values. Furthermore, the sample size of both the control and patient groups was small. Multiple comparisons should be interpreted with caution given the small sample size; therefore, the number of tagged segments within each anatomical region needs to be much larger, and a database with data obtained from many subjects is needed.

## Conclusion

Analysis of 3T tagged MR can non-invasively quantify the transmural degree of left ventricular deformation and demonstrate predominate impairment of subendocardial strain in the chronic ischemic myocardium at rest. Subendocardial C-strain has potential as a sensitive index for the detection of stenotic coronary segment with a diagnostic capability equal to stress/rest SPECT.

## Competing interests

Nagao M.: Bayer Healthcare Japan, Modest, Research Grant; Philips Electronics Japan, Modest, Research Grant

Higuchi K.: Employ by Philips Electronics Japan

Other authors: None

## Authors' contributions

MN carried out the analysis of tagged CMR, participated in the sequence alignment and drafted the manuscript. MH and HH participated in the design of the study and helped to draft the manuscript. YM, TK, and KH carried out the analysis of tagged MRI and performed the statistical analysis. FS, MN, and TM conceived of the study, and participated in its design and coordination.

All authors read and approved the final manuscript.
